# P-MAPA, a Fungi-Derived Immunomodulatory Compound, Induces a Proinflammatory Response in a Human Whole Blood Model

**DOI:** 10.1155/2020/8831389

**Published:** 2020-11-24

**Authors:** Mariana Torrente Gonçalves, Carla Cristina Squaiella-Baptistão, Giselle Pidde, Priscila Hess Lopes, Iseu da Silva Nunes, Denise V. Tambourgi

**Affiliations:** ^1^Immunochemistry Laboratory, Instituto Butantan, São Paulo, Brazil; ^2^Farmabrasilis, Campinas, Brazil

## Abstract

P-MAPA is a complex compound, derived from *Aspergillus oryzae* cultures, that has shown immunomodulatory properties in infection and cancer animal models. Despite promising results in these models, the mechanisms of cellular activation by P-MAPA, suggested to be Toll-like receptor- (TLR-) dependent, and its effect on human immune cells, remain unclear. Using an *ex vivo* model of human whole blood, the effects of P-MAPA on complement system activation, production of cytokines, and the expression of complement receptors (CD11b, C5aR, and C3aR), TLR2, TLR4, and the coreceptor CD14 were analyzed in neutrophils and monocytes. P-MAPA induced complement activation in human blood, detected by increased levels of C3a, C5a, and SC5b-9 in plasma. As a consequence, CD11b expression increased and C5aR decreased upon activation, while C3aR expression remained unchanged in leukocytes. TLR2 and TLR4 expressions were not modulated by P-MAPA treatment on neutrophils, but TLR4 expression was reduced in monocytes, while CD14 expression increased in both cell types. P-MAPA also induced the production of TNF-*α*, IL-8, and IL-12 and oxidative burst, measured by peroxynitrite levels, in human leukocytes. Complement inhibition with compstatin showed that P-MAPA-induced complement activation drives modulation of C5aR, but not of CD11b, suggesting that P-MAPA acts through both complement-dependent and complement-independent mechanisms. Compstatin also significantly reduced the peroxynitrite generation. Altogether, our results show that P-MAPA induced proinflammatory response in human leukocytes, which is partially mediated by complement activation. Our data contribute to elucidate the complement-dependent and complement-independent mechanisms of P-MAPA, which ultimately result in immune cell activation and in its immunomodulatory properties in infection and cancer animal models.

## 1. Introduction

P-MAPA, a compound obtained from the fermentation of *Aspergillus oryzae* fungus cultures [1], developed by Farmabrasilis research network, has shown positive immunomodulatory properties in various pathologic conditions. This compound has a complex composition of magnesium, ammonium, phosphate, linoleic acid, and a 16 kDa protein fraction, with a total molecular weight of 316 kDa [2], and it is obtained in the form of crystals.

Experimental studies have shown that the use of P-MAPA, in the form of crystals, improved the rate of cancer survival, by inhibiting tumor growth in mice and rats bearing Ehrlich ascites tumor (EAT) [3] or bladder cancer [1], respectively. The load of intracellular pathogens was also reduced after P-MAPA treatment, as showed in mice infected with Punta Toro virus [4] or with *Mycobacterium tuberculosis* [1] and also in dogs infected with *Leishmania infantum* [5]. P-MAPA has also been shown to partially inhibit apoptosis in cell lines infected by Zika virus [6]. Recently, the FDA granted P-MAPA the status of orphan drug for the treatment of schistosomiasis, caused by *Schistosoma mansoni* [7]. Still, the mechanism by which P-MAPA activates cells is still unclear.

It has been suggested that the antitumor and pathogen-resistant properties of P-MAPA are related to its immunomodulatory effects, such as the ability to increase, in a dose-dependent manner, the number of bone marrow CFU-GM (colony forming unit-granulocyte/macrophage), as observed in murine models of EAT [3] and *Listeria monocytogenes* infection [8]. It was also reported that treatment of *Leishmania*-infected dogs with P-MAPA crystals increased the percentage of circulating CD8^+^ T lymphocytes, as well as the production of IFN-*γ* and IL-2. Moreover, it induced a decrease of IL-10 levels, produced by peripheral blood mononuclear cells [5].

It has been proposed that P-MAPA interacts with Toll-like receptors (TLR) 2 and 4, as demonstrated by the *in vitro* activation of NF-*κ*B in HEK293 cells expressing human TLR2 or TLR4, but not in cells expressing TLR3, TLR5, TLR7, TLR8, or TLR9 [1]. *In vivo*, P-MAPA also increased the expression of TLR2 in macrophages from *Leishmania chagasi*-infected dogs [8], and of TLR2 and TLR4 in urothelial cells from female rats treated for bladder cancer [1, 9, 10]. Moreover, P-MAPA induced phosphorylation of IKK and p38 MAPK in macrophages from *L. chagasi*-infected dogs, as well as the production of reactive oxygen species (ROS) and nitric oxide (NO), suggesting its interaction with TLR2 [8].

TLR2 and TLR4 are key proinflammatory pattern recognition receptors, which can crosstalk with the complement system, thus reinforcing or controlling inflammation by regulating the expression of cellular receptors and cytokine production [11]. Although inflammation is a natural process during infection and tissue damage, imbalance of the inflammatory processes can lead to depletive consequences, as observed in sepsis or in immunosuppression.

Although much work has been done in animal models, there is no evidence of the effect of P-MAPA in human cells. Moreover, *in vivo* studies have used P-MAPA in the form of insoluble crystals, resuspended in saline buffer or DMSO. In light of the immunomodulatory properties of P-MAPA observed in cell lines and animal models, here we have analyzed the effect of this compound in human whole blood model. The aim was to examine the P-MAPA effect on the complement system and modulation of the surface receptors involved in leukocyte activation during inflammation.

## 2. Materials and Methods

### 2.1. P-MAPA Solubilization and Preparation

P-MAPA was produced and kindly supplied by Farmabrasilis (Campinas, SP, Brazil), in the form of crystals [2]. The crystals were solubilized in 0.25% formic acid in deionized water (for 1 mg of P-MAPA) and then dried under vacuum and resuspended in sterile PBS (8.1 mM Na_2_HPO_4_; 1.5 mM KH_2_PO_4_; 137 mM NaCl; 2.7 mM KCl, pH 7.4). After solubilization, samples of P-MAPA were tested for LPS contamination using PYROGENT™ Plus Gel Clot LAL Assays (Lonza, MD, USA) by the Microbial Control Unity of Butantan Institute (São Paulo, SP, Brazil). Endotoxin contamination was below detection level (0.125 EU/mL), indicating that the results obtained in this study were due to P-MAPA's effect.

### 2.2. Blood and Plasma Collection

This study was approved by the National Commission on Research Ethics under the protocol number CAAE: 02211512.5.0000.0082. Peripheral blood was collected from 6 healthy adult volunteers (21-24 years old) in polypropylene tubes containing lepirudin anticoagulant (Refludan, Celgene, NY, USA) (50 *μ*g/mL), an anticoagulant that does not interfere with complement activation [12, 13].

### 2.3. Complement Activation by P-MAPA

The effect of P-MAPA on the activation of the classical alternative and lectin complement pathways was evaluated *in vitro*, as previously described [14]. Briefly, normal human serum (NHS) obtained from healthy donors was incubated with PBS or P-MAPA at 37°C, for 30 minutes for classical and lectin pathways or for 1 hour for the alternative pathway activation. The residual hemolytic activity was evaluated on sheep erythrocytes for the classical pathway or on rabbit erythrocytes for the alternative pathway. The use of sheep and rabbit erythrocytes was approved by the Animal Ethics Committee from Butantan Institute (CEUA 1145/13). The activation of the lectin pathway was evaluated by ELISA, as described [14].

### 2.4. Stimulation of Human Whole Blood with P-MAPA

The following protocol was based on previous whole blood models for the study of the complement system [13, 15, 16]. Aliquots of fresh blood (72% of total volume of the reaction) were immediately added in sterile polypropylene tubes containing PBS (14% of total volume). Blood samples were then stimulated with PBS (negative control), LPS from *Escherichia coli* (0111:B4, Sigma, MO, USA) (positive control, 100 *μ*g/mL), or P-MAPA (125, 250, 500, or 1000 *μ*g/mL) (14% of total volume) and incubated in water bath at 37°C for 30 minutes. The amount of LPS used in this study was based on previous data showing that high concentrations of LPS (10 to 1000 *μ*g/mL) are necessary to achieve complement activation in lepirudin-treated plasma [15].

After incubation, samples were immediately placed on ice. An aliquot of each experimental treatment was collected for analysis by flow cytometry, and the remaining volume was centrifuged (400*g*, 4°C, 10 min) for plasma collection. EDTA (10 mM) (Sigma) was added to plasma, in order to stop complement activation, and stored at -80°C for further analysis.

### 2.5. Measurement of Complement Factors and Cytokines in Plasma

Plasma collected from human whole blood stimulated with different concentrations of P-MAPA was tested in commercial kits, for measurement of anaphylatoxins C3a/C3a desArg and C5a/C5a desArg, using BD OptEIA Human C3a and C5a ELISA Kits (BD Biosciences) or Cytometric Bead Array (CBA) Human Anaphylatoxin Kit (BD Biosciences), and sC5b-9, using MicroVue sC5b-9 Plus EIA Kit (Quidel Corporation). Cytokines IL-1*β*, IL-6, IL-8, IL-10, IL-12(p70), TNF-*α*, and IFN-*γ* were measured using BD OptEIA Human cytokine ELISA Sets (BD Biosciences) or CBA Human Inflammatory Cytokines Kit (BD Biosciences). All assays were performed according to the manufacturer's instructions.

### 2.6. Analysis of Neutrophil and Monocyte Receptors by Flow Cytometry

After treatment with P-MAPA or controls, blood was incubated with BD FACS Lysing Solution (BD Biosciences, San Jose, CA, USA), for 20 min, at room temperature, for erythrocytes lysis. Samples were centrifuged (720 *g*, 4°C, 10 min), the supernatants were discarded, and cells were washed in FACS buffer (BSA 1%; sodium azide 0.01% in PBS). Cells were then resuspended in FACS buffer containing paraformaldehyde 0.5%, distributed in FACS tubes and stained with anti-CD11b (ICRF44) PE, anti-CD14 (61D3) FITC (both from eBioscience, San Diego, CA, USA), anti-TLR2 (TL2.1) PE, anti-TLR4 (HTA125) PE (both from Life Technologies, San Diego, CA, USA), anti-C3aR (17) FITC, or anti-C5aR FITC (8D6) (both from Santa Cruz Biotechnology, Dallas, TX, USA) at room temperature for 15 minutes. Samples were soon analyzed using BD FACSCanto II flow cytometer, with BD FACSDiVa software, version 4.1 (all from BD Biosciences). Expression of surface receptors was analyzed by gating at neutrophils and monocytes within the total leukocyte population and acquiring 10,000 events (Supplemental Figure 1). The size (FSC-A) and complexity (SSC-A) patterns of cells suggest that LPS or P-MAPA treatments did not induce significant toxicity (Supplemental Figure 1). Data were expressed as median fluorescence intensity (MFI), after subtraction of background MFI from isotype-matched controls (all from Dako, Glostrup, Denmark). Additionally, the effects of solubilized P-MAPA or P-MAPA in the form of crystals were compared, using the whole blood model and analyzing the expression of surface markers in the whole leukocyte population.

### 2.7. Inhibition of Complement Activation by Compstatin

Blood samples collected as in Section 2.4 were preincubated with PBS or compstatin (1 mM) for 10 min at room temperature. Compstatin (Ac-ICVVQDWGHHRCT-NH2) was synthesized at the Butantan Institute (São Paulo, SP, Brazil), according to sequences described previously [13]. Following preincubation with inhibitor or control, blood was then stimulated with LPS (100 *μ*g/mL) or P-MAPA (1000 *μ*g/mL) and incubated for 30 min at 37°C. Once incubation ended, samples were processed for flow cytometry analysis as described in Section 2.6.

### 2.8. Measurement of Reactive Oxygen and Nitrogen Species

Analysis of superoxide and peroxynitrite production by total leukocytes was performed as previously described [17]. Following preincubation of blood samples with compstatin (1 mM) for 10 min at room temperature and stimulation with PBS, LPS, or P-MAPA (described in Section 2.4), aliquots of blood (50 *μ*L) were incubated with 1 *μ*L of dihydroethidium (DHE) or dihydrorhodamine 123 (DHR) (5 *μ*M) (both from Sigma) at 5% CO_2_ for 1 h at 37°C, for DHE, or 30°C, for DHR. Following incubation, erythrocytes were lysed with BD FACS Lysing Solution, according to the manufacturer's instructions, and samples were centrifuged (720 *g*, 4°C, 10 min). Supernatants were discarded and cells were washed in FACS buffer, fixed with 0.5% paraformaldehyde and analyzed by flow cytometry.

### 2.9. Statistics

Statistical analysis was performed using GraphPad Prism 7.00 software (GraphPad Software, CA, USA). Data distribution was checked using the D'Agostino-Pearson normality test. For comparison of three or more groups, one-way ANOVA followed by Tukey's posttest was used, and for comparison between two groups, Student's *t*-test was used. Differences were taken as statistically significant when *p* < 0.05.

## 3. Results

### 3.1. P-MAPA Promotes Complement Activation in Human Whole Blood

The effect of P-MAPA on complement activation was first investigated in normal human sera, using hemolytic assays for the classical and alternative pathways, and ELISA for the lectin pathway. P-MAPA was able to promote a dose-dependent consumption of components from both classical and alternative pathways, inducing about 10-30% reduction of hemolytic activity of NHS with different P-MAPA concentrations (50-500 *μ*g/mL) (Supplemental Figure 2). The lectin pathway was not activated by P-MAPA (data not shown).

**Figure 1:**
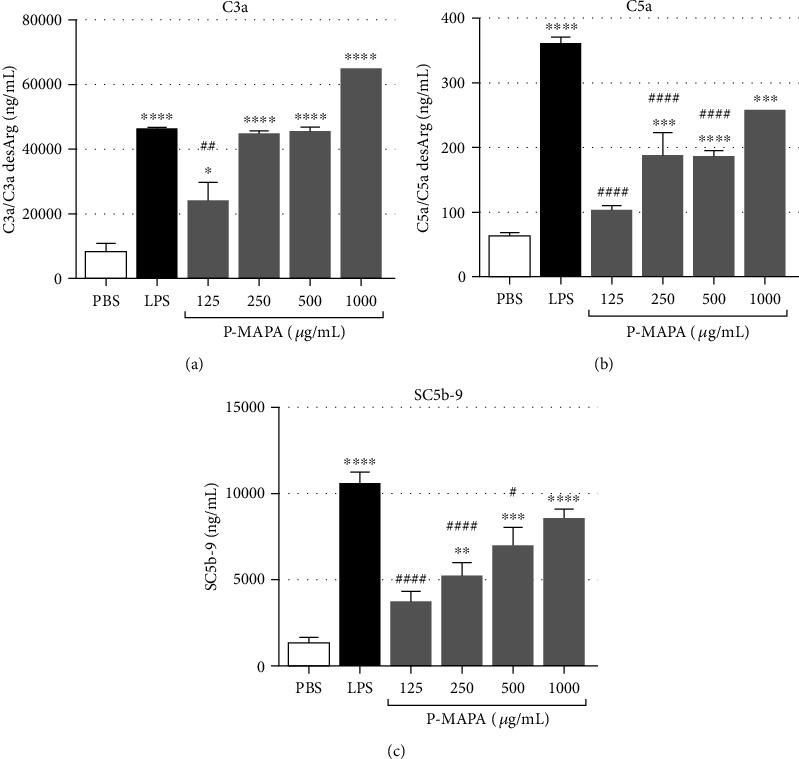
Activation of the complement system by P-MAPA in human whole blood. Complement activation products were measured in plasma from blood samples (720 *μ*L) incubated with PBS, LPS (100 *μ*g/mL), or increasing concentrations of P-MAPA, at 37°C for 30 minutes. (a) Levels of C3a/C3a desArg in plasma, posttreatment, were measured using the OptEIA Human C3a ELISA Kit or BD CBA Anaphylatoxin Kit. (b) Levels of C5a/C5a desArg were measured using the OptEIA Human C5a ELISA or BD CBA Anaphylatoxin Kit. (c) sC5b-9 complex levels in plasma, following incubation with P-MAPA, were measured by EIA detection kit. Data are the mean of three independent experiments, from three different blood donors, performed in duplicate, and the results are expressed as the mean ± SEM. Statistical analysis was performed by one-way ANOVA, complemented with the Tukey test. ^∗^*p* < 0.05, ^∗∗^*p* < 0.01, ^∗∗∗^*p* < 0.001, and ^∗∗∗∗^*p* < 0.0001: significant differences between the mean values with PBS control. ^#^*p* < 0.05, ^##^*p* < 0.01, ^###^*p* < 0.001, and ^####^*p* < 0.0001: significant differences between the mean values with LPS.

The effect of P-MAPA on complement activation in human whole blood was measured in samples treated with increased concentrations of the immunomodulator. Complement activation was detected by the significant increase of anaphylatoxins C3a and C5a in plasma, posttreatment with P-MAPA, compared to PBS-treated blood (Figures 1(a) and 1(b)). To confirm if P-MAPA-induced complement activation leads to the formation of the terminal complement complex (TCC), soluble C5b-9 complex (sC5b-9) was measured in plasma samples from whole blood assays.  Figure 1(c) shows that total complement activation was detected after stimulation of blood with increased concentrations of P-MAPA.

### 3.2. P-MAPA Modulates Surface Receptors in Neutrophils and Monocytes

**Figure 2:**
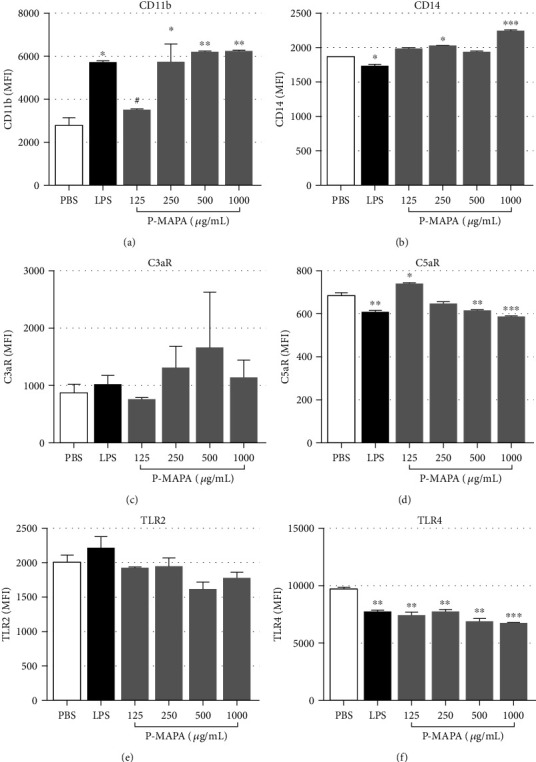
Modulation of surface receptors in monocytes after P-MAPA treatment. After incubation of blood samples with PBS, LPS (100 *μ*g/mL), or P-MAPA (125, 250, 500, or 1000 *μ*g/mL) for 30 minutes at 37°C, erythrocytes were lysed, and leukocytes were stained for flow cytometry analysis. (a) CD11b, (b) CD14, (c) C3aR, (d) C5aR, (e) TLR2, and (f) TLR4 expressions in monocyte population. Data are representative of six separate experiments, from six different donors, performed in duplicate, and the results are expressed as the mean ± SEM. Statistical analysis was performed by one-way ANOVA, complemented with the Tukey test. ^∗^*p* < 0.05, ^∗∗^*p* < 0.01, ^∗∗∗^*p* < 0.001, and ^∗∗∗∗^*p* < 0.0001: significant differences between the mean values with PBS control. ^#^*p* < 0.05, ^##^*p* < 0.01, ^###^*p* < 0.001, and ^####^*p* < 0.0001: significant differences between the mean values with LPS.

**Figure 3:**
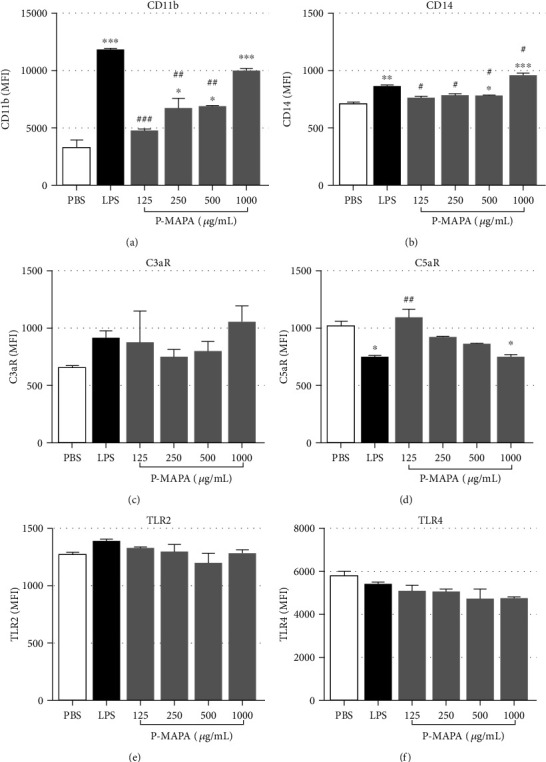
Modulation of surface receptors in neutrophils after P-MAPA treatment. After incubation of blood samples with PBS, LPS (100 *μ*g/mL), or P-MAPA (125, 250, 500, or 1000 *μ*g/mL) for 30 minutes at 37°C, erythrocytes were lysed, and leukocytes were stained for flow cytometry analysis. (a) CD11b, (b) CD14, (c) C3aR, (d) C5aR, (e) TLR2, and (f) TLR4 expressions in neutrophil population. Data are representative of six separate experiments, from six different donors, performed in duplicate, and the results are expressed as the mean ± SEM. Statistical analysis was performed by one-way ANOVA, complemented with the Tukey test. ^∗^*p* < 0.05, ^∗∗^*p* < 0.01, ^∗∗∗^*p* < 0.001, and ^∗∗∗∗^*p* < 0.0001: significant differences between the mean values with PBS control. ^#^*p* < 0.05, ^##^*p* < 0.01, ^###^*p* < 0.001, and ^####^*p* < 0.0001: significant differences between the mean values with LPS.

After blood samples were stimulated with PBS, LPS, or increased concentrations of P-MAPA, erythrocytes were lysed and leukocytes stained for CD11b, CD14, C3aR, C5aR, TLR2, and TLR4 expressions. A 30-minute incubation was sufficient to induce CD11b upregulation by both LPS and P-MAPA stimulations, in monocytes (Figure 2(a)) and neutrophils (Figure 3(a)). CD14 was also upregulated by P-MAPA in both cell types (Figures 2(b) and 3(b)), mainly with the concentration of 1000 *μ*g/mL. C3aR expression (Figures 2(c) and 3(c)) remained unchanged in both cell types after treatments; however, C5aR expression was downregulated in a dose-dependent manner by P-MAPA, in both cell types (Figures 2(d) and 3(d)), with 1000 *μ*g/mL of P-MAPA inducing the same downregulation of LPS. Changes in TLR2 expression were not statistically significant (Figures 2(e) and 3(e)), but TLR4 expression was shown to be decreased in monocyte poststimulation with LPS or P-MAPA, compared to PBS (Figure 2(f)), whereas no change in TLR4 expression was observed in neutrophils (Figure 3(f)). Moreover, Supplemental Figure 3 presents the comparison of the P-MAPA action, in the solubilized or crystal forms (nonsolubilized), in human blood. Data obtained showed similar results in the expression of leukocytes cell surface markers induced by the two P-MAPA forms.

### 3.3. P-MAPA Induces the Production of Proinflammatory Cytokines

**Figure 4:**
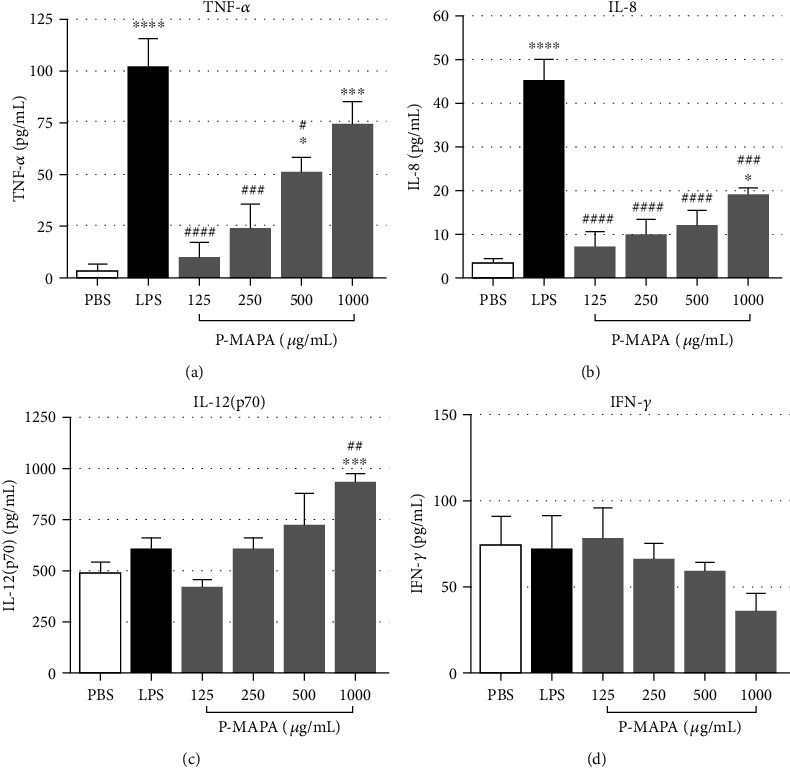
Cytokine production poststimulation of human whole blood with P-MAPA. Cytokines were measured in plasma collected from blood stimulated with PBS, LPS (100 *μ*g/mL), or P-MAPA for 30 minutes at 37°C. (a) TNF-*α* (assay detection limit 7.8 pg/mL), (b) IL-8 (assay detection limit: 3.1 pg/mL), (c) IL-12(p70) (assay detection limit: 7.8 pg/mL), and (d) IFN-*γ* (assay detection limit: 4.7 pg/mL) productions are expressed as the mean ± SEM. Data are the mean of three separate experiments, from three different donors, performed in duplicate, and the results are expressed as concentration of each cytokine per mL of human plasma (pg/mL). Statistical analysis was performed by one-way ANOVA, complemented with the Tukey test. ^∗^*p* < 0.05, ^∗∗^*p* < 0.01, ^∗∗∗^*p* < 0.001, and ^∗∗∗∗^*p* < 0.0001: significant differences between the mean values with PBS control. ^#^*p* < 0.05, ^##^*p* < 0.01, ^###^*p* < 0.001, and ^####^*p* < 0.0001: significant differences between the mean values with LPS.

Having observed the modulation of leucocyte surface receptors within 30 minutes of stimulation with P-MAPA, the levels of cytokines in plasma from blood samples treated with P-MAPA were measured. P-MAPA, at concentrations of 500 and 1000 *μ*g/mL, induced production of TNF-*α* (Figure 4(a)); IL-8 was also detected in a dose-dependent manner, although levels of this cytokine were not as high as of LPS treatment (Figure 4(b)). P-MAPA promoted a significant increase of IL-12(p70) production at the concentration of 1000 *μ*g/mL, compared to PBS and LPS controls (Figure 4(c)). In contrast, levels of IFN-*γ* showed a trend towards a dose-dependent decrease upon treatment with P-MAPA, although these differences were not statistically significant (Figure 4(d)). Although plasma was also tested for the presence of IL-6, IL-1*β*, and IL-10 productions, levels of these cytokines were not detected in any of the experimental treatments (data not shown), likely due to the length of incubation used (30 minutes).

### 3.4. Inhibition of Complement Activation by Compstatin

**Figure 5:**
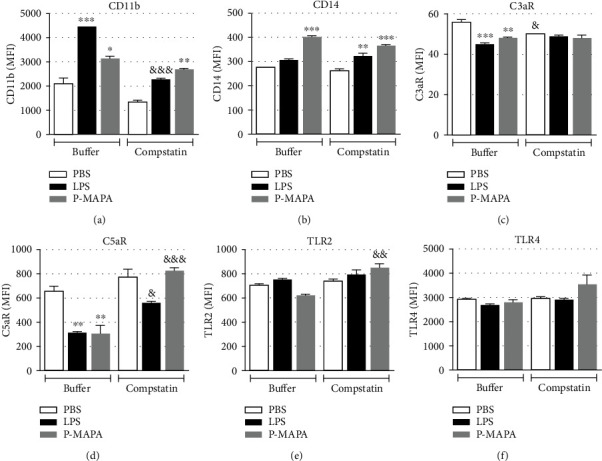
Expression of surface receptors following inhibition of complement activation by compstatin. Blood samples were incubated with compstatin (1 mM) (10 min, RT) ahead of stimulation with PBS, LPS (100 *μ*g/mL), or P-MAPA (1000 *μ*g/mL) for 30 minutes at 37°C. Next, erythrocytes were lysed, and leukocytes were stained for flow cytometry analysis. (a) CD11b, (b) CD14, (c) C3aR, (d) C5aR, (e) TLR2, and (f) TLR4 expressions. Data are representative of three separate experiments, from three different donors, performed in duplicate, and the results are expressed as the mean ± SEM. Comparison between treatments was analyzed using ANOVA and Tukey's posttest. ^∗^*p* < 0.05, ^∗∗^*p* < 0.01, ^∗∗∗^*p* < 0.001, and ^∗∗∗∗^*p* < 0.0001: significant differences between the mean values with PBS control. Comparison between samples treated with compstatin or not (buffer) was analyzed using Student's *t*-test, and significant differences are represented by ^&^*p* < 0.05, ^&&^*p* < 0.01, and ^&&&^*p* < 0.001.

Having shown that P-MAPA could activate the complement system in human blood, we investigated if the complement activation by this compound was responsible for modulation of cellular receptors. Firstly, complement inhibition by compstatin, a protein inhibitor of the C3 protein, was tested in hemolytic assay, in which inhibition of complement activity was successfully achieved after preincubation of sera with 1 mM compstatin (data not shown). Blood samples were then preincubated with compstatin (1 mM) and stimulated with LPS or P-MAPA. For these experiments, the concentration of 1000 *μ*g/mL of P-MAPA was chosen, once this concentration induced statistically significant changes in receptor expression in previous experiments. In these experiments, the expression of cell markers was evaluated in the whole population of leukocytes. Compstatin partially controlled the increased expression of CD11b on LPS blood treated samples, but not on P-MAPA treatment (Figure 5(a)). However, compstatin completely inhibited the downregulation of C5aR by P-MAPA treatment but only exerted a partial effect on its expression by LPS treatment (Figure 5(d)). Moreover, complement inhibition ahead of P-MAPA treatment induced an increase in TLR2 expression (Figure 5(e)).

### 3.5. Leukocytes Activated by P-MAPA Produce Peroxynitrite, Which Is Reduced upon Complement Inhibition

**Figure 6:**
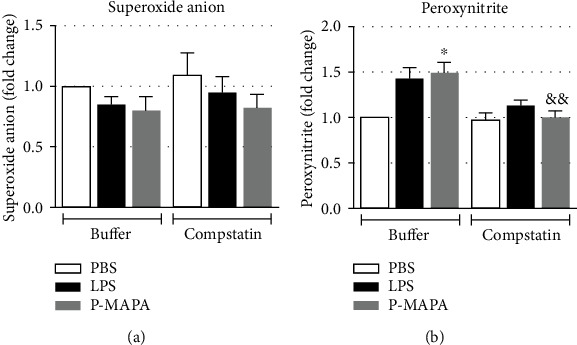
Production of oxygen and nitrogen of reactive species production after complement inhibition and P-MAPA stimulation. Blood samples were preincubated with compstatin (1 mM) (10 min, RT) or left untreated, ahead of stimulation with PBS, LPS (100 *μ*g/ml), or P-MAPA (1000 *μ*g/ml) for 1 h with DHE at 37°C for the detection of superoxide (a) or with DHR at 30°C for detection of peroxynitrite (b), in 5% CO_2_ incubator. Erythrocytes were lysed and samples analysed by flow cytometry. Data are the mean of three separate experiments, from three independent donors, performed in duplicate, and results are expressed as the fold change of median fluorescence intensity (MFI) compared to the control group ± SEM. Comparison between compstatin-treated and compstatin-untreated samples were analysed using the Mann-Whitney test. ^∗^*p* < 0.05: significant differences between the mean values with PBS control. ^&&^*p* < 0.01: significant differences between compstatin-treated and compstatin-untreated groups.

In order to verify if P-MAPA induced the production of reactive oxygen and nitrogen species (ROS and RNS, respectively) in human leukocytes, and if complement activation was involved, blood samples were pretreated or not with compstatin (1 mM), ahead of incubation with LPS or P-MAPA (1000 *μ*g/mL). Following treatments, cells were incubated with substrates DHR or DHE for analysis of production of superoxide and peroxynitrite, respectively, by the whole population of leukocytes. The production of superoxide was below baseline (PBS) levels, poststimulation with LPS or P-MAPA, with no changes promoted by compstatin (Figure 6(a)). In contrast, P-MAPA induced the production of peroxynitrite by leukocytes, and generation of this nitrogen species was reduced in the presence of compstatin (Figure 6(b)).

## 4. Discussion

The immunomodulatory effects of P-MAPA have been described in animal models, but its effects on human cells had never been assessed. Besides, its molecular mechanisms are still poorly understood. P-MAPA was also granted status of orphan drug by the FDA, for the treatment of schistosomiasis [7]; thus, understanding the effect of P-MAPA on immune cells will contribute to novel therapeutic applications of this compound. Here, we reported the first data of P-MAPA treatment in human cells. Specifically, our results demonstrated that P-MAPA can induce complement activation in human blood and modulate surface receptors in neutrophils and monocytes and the production of proinflammatory cytokines by these cells. Also, here we achieved solubilization of P-MAPA crystals with formic acid solution, in contrast with previous studies, where insoluble crystals were resuspended in saline solution or DMSO. Further studies with soluble P-MAPA are required to understand how solubilization alters its structure, but herein we have demonstrated that the most consistent data of surface molecule expressions, which were CD11b, CD14, and C5aR, were similar when comparing solubilized P-MAPA or in the form of crystals (Supplemental Figure 3).

In order to access complement activity in whole blood, we chose a short incubation time and used a lepirudin-based anticoagulant that has no adverse effect on complement activation [13]. A high concentration of LPS was used as a positive control for complement activation in the whole blood model, based on previous data [15]. Similar activation was observed by *in vitro* treatment of whole blood with P-MAPA that produced increased levels of anaphylatoxins C3a and C5a, and of sC5b-9, indicating activation of the terminal complement cascades. Complement activation by P-MAPA was also demonstrated in functional assays of classical, alternative, and lectin pathways. Classical and alternative pathway components were shown to be consumed by P-MAPA in NHS (Supplemental Figure 2). These effects may be related to linoleic acid and magnesium ions in its composition, once that linoleic acid is required for binding of low-density lipoprotein to complement C1q [2, 18]. However, activation of the alternative pathway by P-MAPA may be spontaneous and related with the high concentration of magnesium ions in the compound, as magnesium is an essential cation for activation of this pathway [19].

Anaphylatoxins are proinflammatory mediators that can induce the production of TNF-*α*, IL-1*β*, IL-6, and IL-8 in monocytes [20, 21] and neutrophils [22, 23], upon activation of complement receptors C3aR, CD11b/CD18 (CR3), and C5aR in leukocytes [15, 16, 24, 25]. Indeed, following LPS (positive control) or P-MAPA stimulation of human blood, increased levels of TNF-*α* and IL-8 were found in plasma, as well as increased levels of IL-12 in blood treated with 1000 *μ*g/mL of P-MAPA. Although there are evidences showing that high concentrations of LPS can downmodulate cytokine production or cell activation [26], herein we were still able to observe TNF-*α* and IL-8 release by blood cells treated with LPS in the concentration of 100 *μ*g/mL.

Additionally, flow cytometry analysis showed that, similar to LPS, P-MAPA treatment promoted increase of CD11b and decrease of C5aR expression in neutrophils and monocytes, which indicate activation of these receptors [15, 16, 27, 28], suggesting their involvement in the production of the cytokines found in plasma. Again, despite the high concentration of LPS used as a positive control, we were still able to observe cell activation by LPS in our model, as shown by CD11b upregulation and C5aR downregulation in both cell populations (monocytes and neutrophils).

In addition, inhibition of C3 cleavage by compstatin showed P-MAPA-induced complement activation drives modulation of C5aR, but not of CD11b, suggesting that P-MAPA acts through both complement-dependent and complement-independent mechanisms.

Expression of CD14 also increased following P-MAPA treatment in neutrophils and monocytes, as seen with LPS stimulation, which is known to upregulate CD14 [29, 30]. CD14 works as a coreceptor for TLR4 and TLR2, transferring LPS to TLRs to initiate signaling [31, 32]. Whether P-MAPA binds to CD14 directly is not known. CD14 is involved in triggering oxidative burst in leukocytes [15, 16], alongside complement receptors, TLR2 and TLR4 [33, 34].

Indeed, P-MAPA induced oxidative burst in human leukocytes, as demonstrated by the production of peroxynitrite (OONO^−^), which occurs upon reaction between superoxide anion (O_2_^−^) and nitric oxide (NO) [34]. Therefore, it is possible that the observed decrease in superoxide anion may be a consequence of its chemical reaction with NO, resulting in the formation of peroxynitrite. The oxidative burst had already been observed in neutrophils after only 10 minutes of incubation with *E. coli*, in lepirudin-treated whole blood model [15]. In the referred manuscript, the oxidative burst was evaluated by using dihydrorhodamine 123 (DHR), which readily detects peroxynitrite. Our results are in accordance to a study in which P-MAPA increased ROS production by macrophages from *Leishmania chagasi*-infected dogs [8]. As demonstrated by complement inhibition, reduction in peroxynitrite generation by P-MAPA suggests the compound can promote indirect generation of RNS via complement activation, in human leukocytes.

Studies suggest that P-MAPA directly activates TLR2 and TLR4, as demonstrated by the increased expression of these TLRs in HEK293 cells *in vitro* [1], and in animal models of ovarian [35] and invasive bladder cancer [10], as well as by increased cytosolic levels of TLR-associated downstream signaling molecules MyD88, TRIF, and NF-*κ*B p65 [35]. To this date, there are no studies on how P-MAPA activates TLRs, but it is possible that the lipids present in P-MAPA's composition, such as palmitoleic acid and linoleic acid [2], may be involved in human complement and TLR activation. Of these lipids, palmitoleic acid can enhance TLR4 expression *in vitro* [36] and linoleic acid has been reported to promote phosphorylation of ERK 1/2 and p38 MAPK and increase of IL-8 mRNA in neutrophils after *in vitro* stimulation [37]. These kinases are involved in TLR2 and TLR4 signaling pathways and known to promote neutrophil activation via these receptors [38, 39].

Upregulation of TLR2 and TLR4 by P-MAPA was previously reported in neoplastic tissues [1, 10, 35] and in macrophages from infected animals [8]. In contrast, our study of human neutrophils and monocytes showed no changes in TLR2 expression following P-MAPA treatment, while TLR4 expression was reduced in monocytes. It is possible that significant increase of TLRs could have been observed if our samples were incubated for longer [40, 41]. Furthermore, here we achieved solubilization of P-MAPA crystals with a formic acid solution, in contrast with previous studies, in which P-MAPA was resuspended in saline [35], partially diluted in DMSO [1] or sonicated and resuspended in RPMI-1640 [8]. All these factors could have contributed for contrasting data.

The downregulation of TLR4 expression overserved in monocytes after P-MAPA treatment could be associated with the modulation of CD14. Here, we report that P-MAPA increased the surface expression of CD14 in neutrophils and monocytes. CD14-dependent TLR4 endocytosis has been reported in mononuclear phagocytes and dendritic cells stimulated with LPS for short periods [42]. Interestingly, the authors showed that mature dendritic cells stimulated with TNF or CpG and then treated with LPS expressed higher levels of CD14 surface staining, while lower percentage of surface TLR4, similar to our data. Thus, CD14 may be important for the internalization of TLR4, but CD14 itself may not necessarily be internalized. This mechanism is essential for internalization of TLR4 into the endosome and consequent activation of TRIF/TRAM-dependent signaling pathway, which culminates in the production of type I interferons [42]. Type I interferons are mostly known for their ability to render cells resistant to virus infection, but they have also been implicated in the immune responses against tumor cells [43] and bacterial infections [44]. We thus hypothesize that TLR4 downregulation observed in human blood monocytes treated with P-MAPA could be a consequence of CD14-dependent TLR4 endocytosis, since the direct action of P-MAPA on TLR4 has already been demonstrated [1]. Herein, we did not analyze the production of type I interferons by P-MAPA, but it is possible that the interaction between CD14 and TLR4 may be related to the antiviral, antitumor, and antimicrobial effects previously attributed to P-MAPA treatment [1, 3, 5].

The production of a number of proinflammatory mediators by P-MAPA in human whole blood demonstrates its potential in enhancing the immune response. For instance, the generation of chemoattractants IL-8, C5a, and C3a could potentially drive neutrophils and monocytes to infected or damaged tissues, and TNF-*α* production could also promote inflammation, cell proliferation, and host defense [45]. All these mediators could therefore be related to the previously reported immunomodulatory effects of P-MAPA.

In human blood, IFN-*γ* levels decreased below baseline levels following treatment with P-MAPA. Conversely, in previous reports of *in vivo models*, PBMCs of dogs infected with *L. chagasi* and treated with P-MAPA produced higher levels of IFN-*γ* than untreated animals [5] and high IFN-*γ* levels were present in urinary bladder neoplastic tissue [9, 10] and ovarian carcinoma of rats treated with P-MAPA [35]. IFN-*γ* is considered to be mainly produced by NK cells and CD4^+^ T cells, although its production has been also attributed to other cell types [46]. Considering the short period of our model, it is reasonable to suggest that basal levels of IFN-*γ*, detected in plasma, might be derived from NK cells. These cells usually produce IFN-*γ* in response to IL-12 released by activated monocytes/macrophages [47]. Intriguingly, in our model, the marked impairment of IFN-*γ* release in P-MAPA-treated blood was not accompanied by a decrease in IL-12 production. Thus, it seems that P-MAPA probably induced a regulation mechanism of IFN-*γ* release. TGF-*β* is a known negative regulator of IFN-*γ* release [47], but it was not increased by P-MAPA in our model (data not shown). Another possible explanation for the decrease of IFN-*γ* is the short period of incubation (30 minutes). It is possible that this is a temporary effect, which could be reversible with longer periods of treatment, as already demonstrated in animal models [5, 9, 10, 35]. Thus, the effects of P-MAPA in the release of IFN-*γ* in our model remain to be investigated.

Altogether, our findings show that P-MAPA induces complement activation, with consequent release of C3a and C5a, and production of proinflammatory mediators, namely, TNF-*α*, IL-8, IL-12, and oxidative burst in human leukocytes. P-MAPA also modulates CD11b, CD14, and C5aR expressions in neutrophils and monocytes, and TLR4 in monocytes. Some of these effects are partially mediated by the complement system, while others are probably induced via TLR activation. Our data contribute to elucidate the complement-dependent and complement-independent mechanisms of P-MAPA, which ultimately result in a proinflammatory profile. This promotes immune cell activation, resulting in the previously shown immunomodulatory properties of P-MAPA in infection and cancer animal models.

## Data Availability

The data used to support the findings of this study are included within the article.

## Supplementary Materials

Supplementary Materials Supplemental Figure 1 flow cytometry gating strategy for neutrophils and monocytes. Representative forward/side scatter plot showing (A) the total leukocyte population and (B) within this gate, identification of neutrophils and monocytes. Supplemental Figure 2 effect of P-MAPA on the complement pathways. Samples of normal human serum (NHS) were incubated with PBS or P-MAPA at 37°C, for 30 minutes for classical (A) or for 1 hour for alternative pathway (B). The residual hemolytic activity relative to the PBS control was evaluated on sheep erythrocytes for the classical pathway (A) or on rabbit erythrocytes for the alternative pathway (B) and the absorbance of the supernatants determined at *λ* = 414 nm. Data were expressed as the mean ± standard deviation of duplicates, representative of 3 independent experiments. Statistical analysis performed by one-way ANOVA, complemented with the Tukey test. ^*^*p* < 0.05, ^**^*p* < 0.01, and ^***^*p* < 0.001: statistically significant difference in relation to the control (PBS). ^¢^*p* < 0.05 and ^¢¢^*p* < 0.01: statistically significant difference between treatments with P-MAPA. Supplemental Figure 3 eEffect of solubilized and non-solubilized P-MAPA on the expression of surface markers in leukocytes. Human blood samples were treated with PBS, P-MAPA (1000 *μ*g/mL) solubilized or not, or LPS (100 *μ*g/mL), for 5 minutes, at room temperature, followed by incubation for 30 minutes at 37°C. After incubation, leukocytes were collected and analyzed for expression of surface markers. (A) Expression of CD11b. (B) Expression of CD14. (C) Expression of TLR2. (D) Expression of TLR4. (E) C3aR expression. (F) C5aR expression. Data expressed as the mean ± standard deviation of duplicates from 2 independent experiments. MFI: median fluorescence intensity. Statistical analysis performed by one-way ANOVA, complemented with the Tukey test. ^*^*p* < 0.05, ^**^*p* < 0.01, ^***^*p* < 0.001: statistically significant difference in relation to PBS. ^#^*p* < 0.05 and ^##^*p* < 0.01: statistical difference in relation to LPS. ^¢¢^*p* < 0.01: statistically significant difference between treatments with P-MAPA.
