# Supplementary material for: P-MAPA, a Fungi-Derived Immunomodulatory Compound, Induces a Proinflammatory Response in a Human Whole Blood Model
Source: Mediators Inflamm. 2020 Nov 24;2020:8831389. doi: 10.1155/2020/8831389 (PMC7707968; doi:10.1155/2020/8831389)

**Supplemental Figure 1**


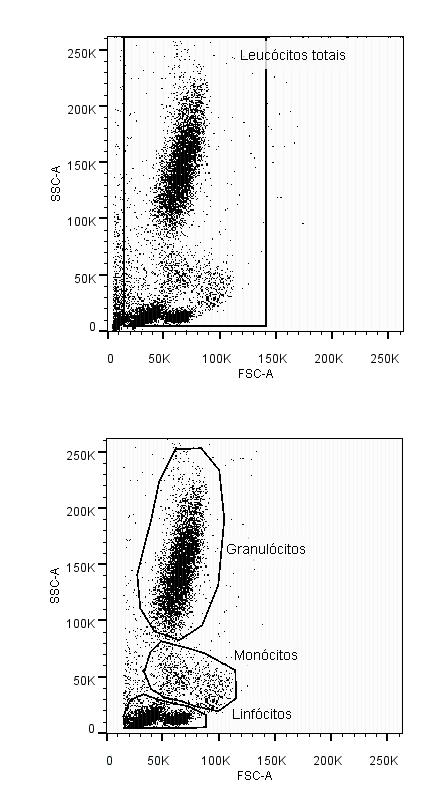

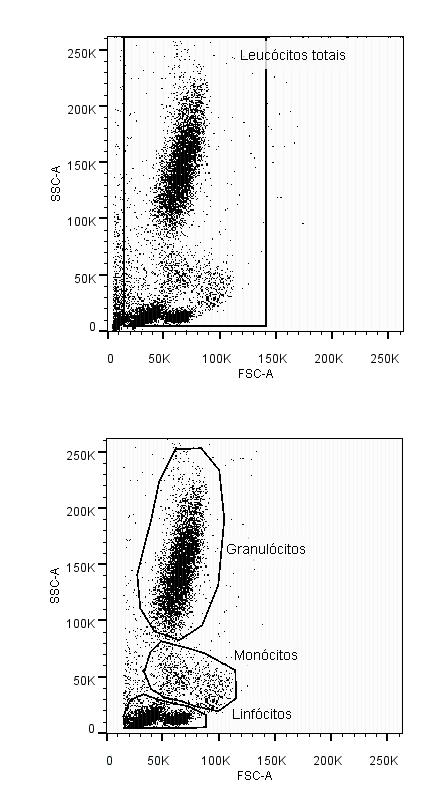


Total leukocytes

Neutrophils

Monocytes

Lymphocytes

**Supplemental Figure 2**

**
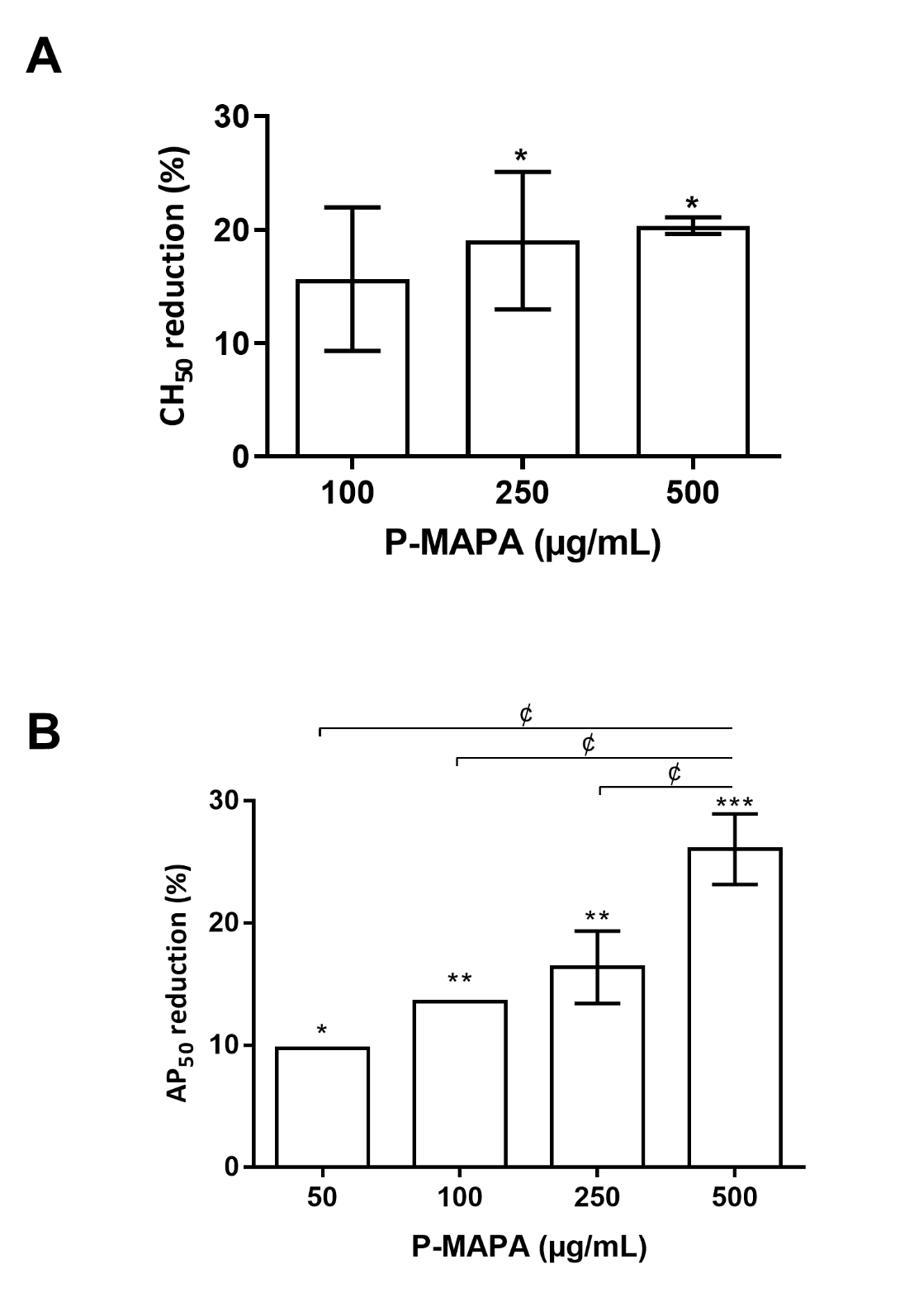
**

**Supplemental Figure 3**


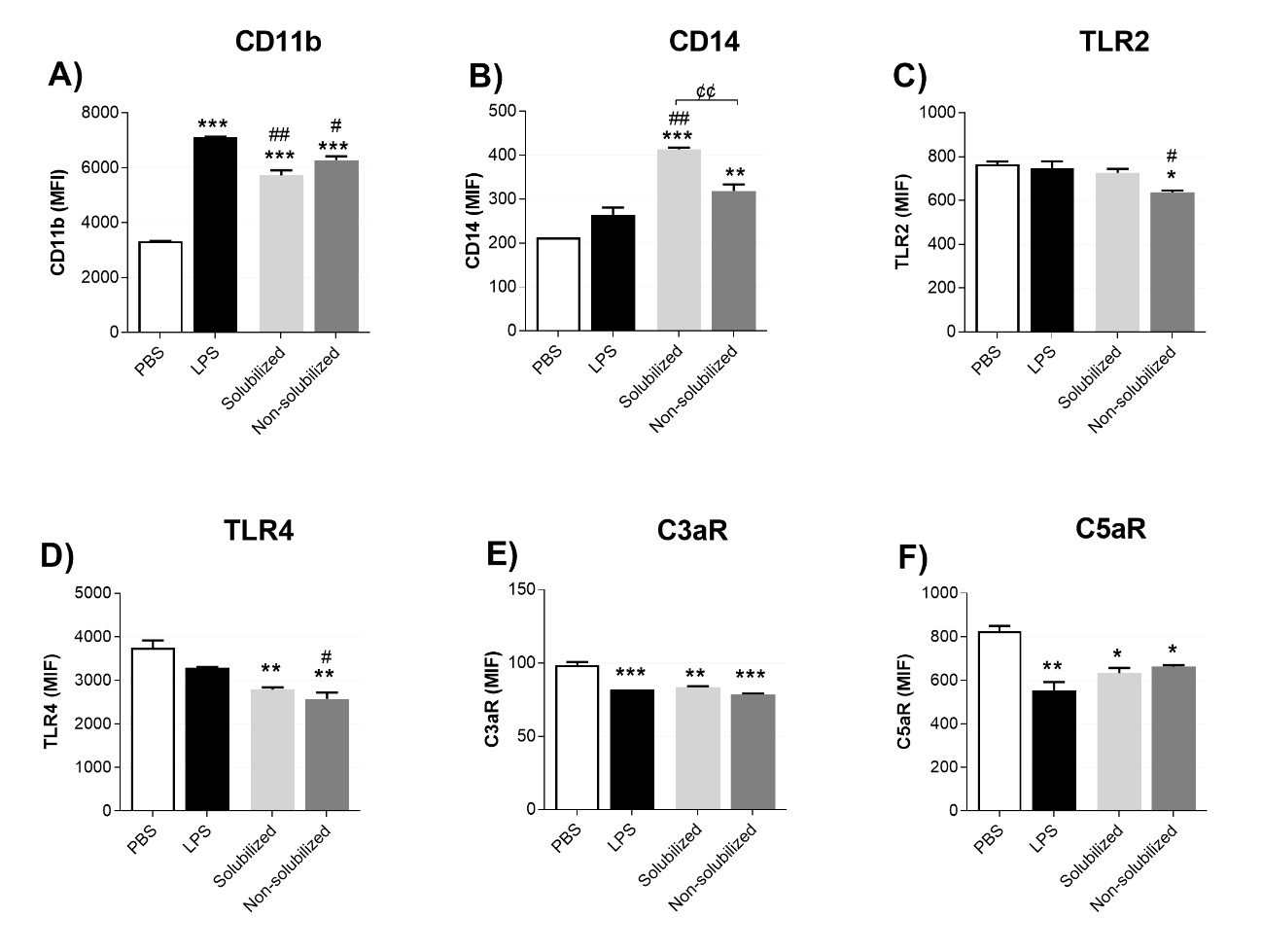

Supplement: Supplementary Materials — Supplemental Figure 1 flow cytometry gating strategy for neutrophils and monocytes. Representative forward/side scatter plot showing (A) the total leukocyte population and (B) within this gate, identification of neutrophils and monocytes. Supplemental Figure 2 effect of P-MAPA on the complement pathways. Samples of normal human serum (NHS) were incubated with PBS or P-MAPA at 37°C, for 30 minutes for classical (A) or for 1 hour for alternative pathway (B). The residual hemolytic activity relative to the PBS control was evaluated on sheep erythrocytes for the classical pathway (A) or on rabbit erythrocytes for the alternative pathway (B) and the absorbance of the supernatants determined at λ = 414 nm. Data were expressed as the mean ± standard deviation of duplicates, representative of 3 independent experiments. Statistical analysis performed by one-way ANOVA, complemented with the Tukey test. *p < 0.05, **p < 0.01, and ***p < 0.001: statistically significant difference in relation to the control (PBS). ¢p < 0.05 and ¢¢p < 0.01: statistically significant difference between treatments with P-MAPA. Supplemental Figure 3 eEffect of solubilized and non-solubilized P-MAPA on the expression of surface markers in leukocytes. Human blood samples were treated with PBS, P-MAPA (1000 μg/mL) solubilized or not, or LPS (100 μg/mL), for 5 minutes, at room temperature, followed by incubation for 30 minutes at 37°C. After incubation, leukocytes were collected and analyzed for expression of surface markers. (A) Expression of CD11b. (B) Expression of CD14. (C) Expression of TLR2. (D) Expression of TLR4. (E) C3aR expression. (F) C5aR expression. Data expressed as the mean ± standard deviation of duplicates from 2 independent experiments. MFI: median fluorescence intensity. Statistical analysis performed by one-way ANOVA, complemented with the Tukey test. *p < 0.05, **p < 0.01, ***p < 0.001: statistically significant difference in relation to PBS. #p < 0.05 and ##p < 0.01: statistical d [file 8831389.f1.docx]
